# A nomogram for predicting the risk of mortality in patients with acute pancreatitis and Gram-negative bacilli infection

**DOI:** 10.3389/fcimb.2022.1032375

**Published:** 2022-11-10

**Authors:** Jia Yan, Huang Yilin, Wu Di, Wang Jie, Wang Hanyue, Liu Ya, Peng Jie

**Affiliations:** Department of Gastroenterology, Xiangya Hospital, Central South University, Changsha, China

**Keywords:** severe acute pancreatitis, carbapenem-resistant Gram-negative bacilli, septic shock, predictive model, nomogram

## Abstract

**Objective:**

Gram-negative bacilli (GNB) are common pathogens of infection in severe acute pancreatitis (SAP), and their occurrence increases the mortality of SAP. Early identification of SAP severity and prognosis is of great significance to SAP treatment. This study explored risk factors for mortality in patients with SAP and GNB infection and established a model for early prediction of the risk of death in GNB-infected SAP patients.

**Methods:**

Patients diagnosed with SAP from January 1, 2016, to March 31, 2022, were included, and their baseline clinical characteristics were collected. Univariate logistic regression analysis was performed to screen for death related variables, and concurrently, a Boruta analysis was performed to identify potentially important clinical features associated with mortality. The intersection of the two results was taken for further multivariate logistic regression analysis. A logistic regression model was constructed according to the independent risk factor of death and then visualized with a nomogram. The performance of the model was further validated in the training and validation cohort.

**Results:**

A total of 151 patients with SAP developed GNB infections. Univariate logistic regression analysis identified 11 variables associated with mortality. The Boruta analysis identified 11 clinical features, and 4 out of 9 clinical variables: platelet counts (odds ratio [OR] 0.99, 95% confidence interval [CI] 0.99–1.00; p = 0.007), hemoglobin (OR 0.96, 95% CI 0.92–1; p = 0.037), septic shock (OR 6.33, 95% CI 1.12–43.47; p = 0.044), and carbapenem resistance (OR 7.99, 95% CI 1.66–52.37; p = 0.016), shared by both analyses were further selected as independent risk factors by multivariate logistic regression analysis. A nomogram was used to visualize the model. The model demonstrated good performance in both training and validation cohorts with recognition sensitivity and specificity of 96% and 80% in the training cohort and 92.8% and 75% in the validation cohort, respectively.

**Conclusion:**

The nomogram can accurately predict the mortality risk of patients with SAP and GNB infection. The clinical application of this model allows early identification of the severity and prognosis for patients with SAP and GNB infection and identification of patients requiring urgent management thus allowing rationalization of treatment options and improvements in clinical outcomes.

## Introduction

Acute pancreatitis (AP), an acute inflammatory disease caused by destruction of acinar cells that perform exocrine function of the pancreas, is one of the most common diseases of the digestive system requiring emergency treatment. Its global incidence is on the rise. The overall mortality rate is about 5% (Mederos et al., 2021). About 20% of patients develop severe acute pancreatitis (SAP) with persistent organ failure as the main manifestation, and the fatality rate is as high as 30% to 50% (Garg and Singh, 2019). In patients with SAP, in addition to the high risk of death caused by systemic inflammatory response syndrome and organ failure during the early stages of the disease, local and/or systemic infectious complications and related organ failure during the middle and late stages of the disease compose the second death peak, and gradually become the main cause of death (Johnson and Abu-Hilal, 2004).

Patients with AP are at high risk of infection due to the overuse of early anti-inflammatory and antimicrobial drugs, and infection is a major complication that jeopardizes the course of AP (Moka et al., 2018). Disruption of the intestinal barrier and translocation of intestinal bacteria in patients with AP has been recognized as the main cause of necrotizing infection in pancreatitis. In AP, bacterial and fungal infections resulting from predominantlyPatients with necrotizing Gram-negative bacteria (GNB) can occur in the abdominal cavity, and pancreatic infection is present in approximately one-third of patients with acute necrotizing pancreatitis (van Brunschot et al., 2012). Patients with necrotizing pancreatitis and infection may have more than twice the mortality rate of uninfected group (Baron et al., 2020). Infectious necrotizing pancreatitis (INP) has been identified as a determinant of mortality in acute pancreatitis study (Petrov et al., 2010). Additionally, approximately one third of patients with AP have extra-pancreatic infections (EPI), such as bacteremia, pneumonia, and urinary tract infections (Bakker et al., 2013). Complications of extra pancreatic infections are also associated with increased mortality (Brown et al., 2014). EPIs are considered to be iatrogenic infections acquired by intestinal bacterial translocation into the systemic circulation or through percutaneous drainage tubes, venous catheters, urinary catheters, and others (Wu et al., 2008). EPI have received more and more attention in recent years. In the process of AP treatment, EPI also needs to be actively evaluated and treated.

Despite the improvements in diagnostic and treatment techniques, the high mortality rate for AP, especially SAP, has not declined over the past decade (Lankisch et al., 2015). Early and accurate assessment of the severity and prognosis of SAP, especially when it occurs with a concurrent infection, is of great significance for clinicians to pay attention to the condition of SAP and to prescribe more effective treatment measures for patients whose condition might worsen. Related research mainly focuses on necrotizing pancreatitis and drug-resistant bacterial infection. Few studies exploring mortality prediction in patients with AP and GNB infection are available. Herein, we used machine learning methods to develop a visual and easy-to-use nomogram to predict the risk of death in patients with AP and GNB infection.

## Methods

### Study design and patients

A retrospective cohort study enrolled cases of moderate SAP and SAP patients confirmed to have infections with GNB and collected relevant clinical data from January 1, 2016, to March 31, 2022, in Xiangya Hospital, Central South University, a 3500-bed tertiary care teaching hospital. At the initial admission, all patients were assessed and managed *via* the multidisciplinary team, including pancreatic surgeons, emergency physicians, gastroenterology physicians, and intensive care unit physicians according to the latest international guidelines (Crockett et al., 2018).

In our cohort, diagnosis and classification of AP were based on the criteria of revised Atlanta classification (Banks et al., 2013). Criteria of etiology are listed: (1) biliary: choledocholithiasis or cholelithiasis by enhanced computed tomography; (2) hypertriglyceridemia: triglyceride >1000 mg/dl; and/or (3) alcoholic: drinking > 50 g/d for at least one year. Patients who were older than 18 years, diagnosed with AP according to Atlanta criteria, and had GNB infection in the advanced period (disease duration > 2 weeks) would also be enrolled in our study. Patients who were younger than 18 years or older than 75 years, diagnosed with mild acute pancreatitis, pregnant, complicated with malignant tumor, and/or infected before the admission to our hospital were excluded.

Eventually, 151 patients were enrolled for further analysis, and they were randomly allocated as a ratio of 7:3 to training and validation cohorts (Zhang et al., 2020). The variable analysis and model establishment were conducted based on the whole data set and further evaluated in the training and validation cohorts, respectively.

### Clinical data collection

Clinical characteristics included general demographic information, etiology, comorbidity, type of acute pancreatitis (recurrent acute pancreatitis [RAP], severe acute pancreatitis), site of infections, bacteria species, laboratory indices, mortality, antibiotic therapy, complications, and the detailed information on admission. Missing data < 20% were subsequently submitted to missing value imputation.

The infection was diagnosed on the basis of clinical presentation and positive culture features according to the criteria of the Centers for Disease Control (Horan et al., 2008). The date of the first positive sample collection was considered as the initiation of the infection. The relevant laboratory data within the initial 24 h after the first positive culture sample were collected, and the worst data were used in the analysis if two or more identical detecting items existed. Commonly used prophylactic anti-infective drugs included broad-spectrum antibiotics (3^rd^/4^th^ generation cephalosporins and quinolones, carbapenems, and combined antibiotics). In addition, antibiotic therapy was administered for “suspected” infections until further positive culture and drug susceptibility results were available for the selection of a targeted therapy. In general, the whole antibiotic procedure lasted approximately 5 days, including the frequently used drugs carbapenems, tigecycline, penicillin/β-lactamase inhibitors, and their combination.

Microbiological identification of drug resistance was performed using the VITEK-2 system (BiomeRieux, Marcy L’etoile, France). Drug susceptibility and minimum inhibitory concentrations were determined using the Kerby–Bauer disk diffusion and agar dilution methods, respectively. Carbapenem resistance (CR) was defined as acquired insensitivity to meropenem or imipenem; minimum inhibitory concentration ≥ to 2 mg/L (Wu et al., 2020).

Clinical outcomes were divided into mortality and survival according to death event during the time of hospitalization.

### Ethics statement

Because of the retrospective nature of this cohort study, the Institutional Review Boards of Xiangya Hospital (no. 202105092) waived the need for direct patient enrollment and informed consent. Information was gathered from electrical medical systems in an anonymous manner in which all authors could ensure the confidentiality of patient data. The study was strictly performed in accordance with the Declaration of Helsinki.

### Feature analysis and selection

#### Using logistic regression analysis

Variables were subjected to univariate logistic regression analysis to calculate odds ratio (OR) and evaluated whether the characteristic factor was significant.

#### Based on random forest

Concurrently, to clarify the exact clinical characteristics concerning with mortality at a probability excelling random, a Boruta analysis was conducted to remove unusable features that could create unnecessary noise. The Boruta analysis is a random forest-based feature selection algorithm (Kursa, 2014) in which the basic logic is to evaluate the importance of each feature variable through a loop method by copying the original feature set and randomly mixing each feature value to construct a shadow feature with randomness; after interactive calculation, the importance of the scores of the original and shadow features were compared, so as to filter out the optimal feature set that can be used for modeling. The final result uses the shadow Max as the screening index. When the feature variable score was greater than the shadow Max, the feature was considered important and accepted; otherwise, the variable was rejected, the remaining variables with importance scores around shadow Max value were divided into a tentative group. All Boruta analyses were performed using the Boruta package version 6.0.0 using the default parameters (ntree = 500, max Runs = 500, p = 0.01).

### Development and validation of a logistic regression model

The intersection of variables with statistical significance that were sifted out in the univariate logistic regression analysis and the important variables distinguished by Boruta analyses were selected. Successively, the intersected variables were taken to multivariate logistic regression analysis to determine independent predictors of mortality. Furthermore, a logistic regression model was established based on independent predictors, and a visual version was constructed through the utilize of a nomogram. Besides, we assessed the model in the training cohort (n = 105) and validation cohort (n = 46), which was determined by the ratio of the patients enrolled into the training cohort and validation cohort in the previous study (Zhang et al., 2020). Predictive performance was assessed by discrimination and calibration.

### Statistical analysis

Other missing values were interpolated using the mice package (3.14.0) of R software for multiple imputation (Hayati Rezvan et al., 2015). Normally, distributed continuous variables were expressed as mean ± standard deviation (SD) and compared using Student’s t-test, and non-normally distributed continuous variables were expressed as median (inter-quartile range [IQR]) and were compared using the Wilcox Sum Rank test. Categorical variables were expressed as count and percentage, which were compared using a chi-squared or Fisher’s exact test.

The intersection of variables with statistical significance sifted out in the univariate logistic regression analysis and the important variables distinguished by Boruta analyses were selected. The intersected variables selected in the previous steps were taken to multivariate logistic regression analysis, to identify the ones with P < 0.05 and construct a clinical predictive model. Furthermore, a receiver operating characteristic curve (ROC) was conducted to measure the predictive accuracy of the developed model, and the area under the receiver operating characteristic curve (AUC) was calculated. Finally, this model was assessed using a bootstrap-based calibration process (Wan et al., 2022) to evaluate the relationship between the actual and predicted probabilities of mortality. The statistical analyses mentioned above were performed using the RMS Version 6.2-0 and pROC Version 1.18.0 R package.

All statistical analyses were performed using R software version4.1.2 (www.r-project.org). P-value < 0.05 was considered statistically significant among the relevant analyses.

## Results

### Baseline characteristics of patients

A total of 151 patients with moderate SAP and SAP developed GNB infection with a mortality rate of 25.8% (39 of 151) and a median age of 48 years over a 6-year period. The ratio of male and female were 70.9% (n = 107) and 29.1% (n = 44), respectively. Hypertriglyceridemia (n = 64, 42.4%) and gallstones (n = 39, 25.8%) were the main etiologies in our cohort. And 23 patients were diagnosed with RAP (15.2%). The most common comorbidities included diabetes (21.2%) and hypertension (20.5%). SAP accounted for 53.6% of the total cases. The pancreas (peri) (64.9%) was the most common site (64.9%) of infection, and *Klebsiella pneumonia* (29.8%) was the most common pathogen. Of the 151 patients, a total of 49% had polymicrobial infections, 51% had multiple site infections, 33.1% had fungal infections, and 30.5% had Gram-positive pathogen infections. Among the Gram-negative pathogens, 47.5% were carbapenem-resistant. Broad-spectrum antibiotics (33.8%) were the most common preventive anti-infective drugs followed by combined antibiotics (30.5%) and carbapenems (29.8%). Carbapenems (35.1%) and carbapenem–tigecycline (29.1%) were the most commonly used treatment protocols for confirmed infection. The incidence of pancreatic leakage and intestinal fistula were 16.6% and 13.9%, respectively. More than one-quarter (31.8%) of patients developed respiratory failure and required mechanical ventilation support, and 17.2% of patients required vasoactive drugs to maintain blood pressure due to septic shock. A comparison of clinical data and laboratory variables between the survival and mortality groups of 151 patients with SAP and GNB infection is shown in **Table 1**. Among the 151 patients who met the inclusion criteria, 105 patients were included in the training cohort and 46 included in the validation cohort, and no significant differences in baseline clinical characteristics between the training and validation cohorts were found (**Supplementary Table 1**). The mortality rates of these two cohorts were 23.8% (25 of 105) and 30.4% (14 of 46), respectively. No significant difference in baseline characteristic between the training and validation cohorts were found.

**Table 1:** Clinical characteristics and comparison between survival and mortality of 151 AP patients with GNB infections.

Variables [n (%), median (IQR) or mean ± SD]	Total (n =151)	Survival (n =112)	Death (n = 39)	p-value
Gender (Male)	107 (70.9)	78 (69.6)	29 (74.4)	0.724
Age, years	48.44 (12.53)	47.54 (12.49)	51.03 (12.45)	0.136
Comorbidity				
Hypertension	31 (20.5)	21 (18.8)	10 (25.6)	0.492
Diabetes	32 (21.2)	25 (22.3)	7 (17.9)	0.728
Hepatitis	4 (2.6)	2 (1.8)	2 (5.1)	0.274
Etiology				
Gallstones	39 (25.8)	26 (23.2)	13 (33.3)	0.011
Hypertriglyceridemia	64 (42.4)	53 (47.3)	11 (28.2)	
Alcoholism	18 (11.9)	8 (7.1)	10 (25.6)	
Idiopathic	3 (2.0)	3 (2.7)	0 (0.0)	
Others	27 (17.9)	22 (19.6)	5 (12.8)	
RAP	23 (15.2)	21 (18.8)	2 (5.1)	0.075
SAP	81(53.6)	46 (41.1)	35 (89.7)	<0.001
Enteral nutrition, days	2.00 (2.00, 5.00)	2.00 (2.00, 4.50)	2.50 (2.00, 5.00)	0.905
PH	7.44 (7.39, 7.48)	7.45 (7.42, 7.48)	7.40 (7.33, 7.48)	0.031
SCr (mmol/L)	66.80 (52.95, 100.75)	62.40 (51.62, 76.38)	218.60 (79.85, 364.70)	<0.001
ALB (g/L)	29.92 (4.76)	30.20 (4.94)	29.09 (4.17)	0.211
TBil (mmol/L)	15.70 (9.60, 26.87)	14.30 (9.17, 20.48)	24.95 (17.25, 67.38)	<0.001
NE (×10^9^/L)	8.20 (5.60, 12.50)	8.00 (5.57, 12.33)	8.60 (6.00, 14.75)	0.563
LYMPH (×10^9^/L)	0.90 (0.60, 1.20)	0.90 (0.60, 1.20)	0.80 (0.50, 1.20)	0.413
PLT(×10^9^/L)	271.99 (132.69)	294.94 (131.79)	206.10 (112.93)	<0.001
Hb (g/L)	88.13 (17.75)	91.79 (17.37)	77.62 (14.44)	<0.001
PCT (ng/L)	1.00 (0.28, 3.71)	0.45 (0.19, 1.75)	6.46 (1.86, 21.34)	<0.001
Temperature,℃	39.00 (38.00, 39.50)	38.95 (37.95, 39.42)	39.20 (38.80, 39.50)	0.06
Carbapenem resistance	69 (45.7)	33 (29.5)	36 (92.3)	<0.001
Septic shock	26 (17.2)	4 (3.6)	22 (56.4)	<0.001
Mechanical ventilation	48 (31.8)	19 (17.0)	29 (74.4)	<0.001
The first infection site				0.012
Pancreas (peri)	98(64.9)	80(71.4)	18(46.1)	
Bloodstream	16 (10.6)	9 (8.0)	7 (17.9)	
Lung	28 (18.5)	15 (13.4)	13 (33.3)	
Urinary system	2 (1.3)	2 (1.8)	0 (0.0)	
Others	7 (4.6)	6 (5.4)	1 (2.6)	
The first infection strain				<0.001
Escherichia coli	32 (21.2)	29 (25.9)	3 (7.7)	
Klebsiella ozaena	45 (29.8)	28 (25.0)	17 (43.6)	
Acinetobacter baumannii	33 (21.9)	18 (16.1)	15 (38.5)	
Pseudomonas aeruginosa	10 (6.6)	9 (8.0)	1 (2.6)	
Others	31 (20.5)	28 (25.0)	3 (7.7)	
Polymicrobial infections	74 (49.0)	50 (44.6)	24 (61.5)	0.103
Concurrent GPB infection	46 (30.5)	35 (31.2)	11 (28.2)	0.878
Fungal infection	50 (33.1)	34 (30.4)	16 (41.0)	0.307
Prophylactic anti-infection				0.3
Wide-spectrum antibiotics	51 (33.8)	38 (33.9)	13 (33.3)	
Carbapenem	45 (29.8)	33 (29.5)	12 (30.8)	
Combined antibiotics	46 (30.5)	32 (28.6)	14 (35.9)	
Antibiotic therapy				0.013
Carbapenem (high-dose, extended-infusion)	53 (35.1)	42 (37.5)	11 (28.2)	
Carbapenem and tigecycline	44 (29.1)	26 (23.2)	18 (46.2)	
Penicillins/β-lactamase inhibitors	29 (19.2)	27 (24.1)	2 (5.1)	
Carbapenem and penicillins/β-lactamase inhibitors	21 (13.9)	14 (12.5)	7 (17.9)	
Tigecycline	2 (1.3)	1 (0.9)	1 (2.6)	
Carbapenem and sulfonamides	1 (0.7)	1 (0.9)	0 (0.0)	
Tigecycline and polymyxin	1 (0.7)	1 (0.9)	0 (0.0)	
Pancreatic leakage	25 (16.6)	18 (16.1)	7 (17.9)	0.983
Intestinal leakage	21 (13.9)	12 (10.7)	9 (23.1)	0.098

IQR, interquartile ranges; SD, standard deviation; RAP, recurrent acute pancreatitis; SAP, severe acute pancreatitis; PH, potential of hydrogen; SCr, serum creatinine; ALB, albumin; TBil, total bilirubin; NE, neutrophilic granulocyte; LYMPH, lymphocyte; PLT, platelet; Hb, hemoglobin; PCT, procalcitonin; Concurrent GPB infection, Concurrent Gram-positive bacterial infection.

### Univariate logistic regression analysis for statistically significant parameters

Risk factors for death in patients with AP and GNB infection were analyzed by univariate logistic regression, and the results are shown in **Table 2**. Eleven variables, including creatinine, bilirubin, platelets, hemoglobin, carbapenem resistance, septic shock, SAP, therapeutic agents, mechanical ventilation, and the first site of infection were found to be significantly associated with mortality.

**Table 2:** Univariate logistic regression analysis based on baseline characteristics in the survival group and death group.

Variables	OR	95% CI	p-value
Age	1.02	0.99 - 1.05	0.137
Male	1.26	0.57 - 2.99	0.577
Etiology	1.078	0.813-1.428	0.603
RAP	0.23	0.04 - 0.85	0.058
SAP	12.55	4.63 - 44.16	< 0.001
Hypertension	1.49	0.61 - 3.48	0.361
Diabetes	0.76	0.28 - 1.86	0.566
Hepatitis	2.97	0.35 - 25.50	0.284
Temperature	1.44	1.01 - 2.12	0.052
PH	0.29	0.03 - 2.64	0.266
SCr	1.01	1.00 - 1.01	< 0.001
ALB	0.95	0.87 - 1.03	0.211
TBil	1.01	1.00 - 1.02	0.016
NE	1	0.95 - 1.03	0.819
LYMPH	0.84	0.42 - 1.10	0.551
PLT	0.99	0.99 - 1.00	< 0.001
PCT	1.03	1.01 - 1.06	0.009
Hb	0.94	0.91 - 0.97	< 0.001
The first infection strain	0.891	0.683-1.162	0.395
Polymicrobial infections	1.98	0.95 - 4.25	0.071
The first infection site	1.316	1.02-1.697	0.035
Fungal infection	1.6	0.74 - 3.39	0.225
Concurrent GPB infection	0.86	0.38 - 1.90	0.722
Carbapenem resistance	28.73	9.52 - 125.01	< 0.001
Prophylactic anti-infection	1.33	0.888-1.992	0.166
Antibiotic therapy	1.293	1.025-1.632	0.03
Eternal nutrition	0.95	0.86 - 1.02	0.251
Septic shock	34.94	11.75 - 131.18	< 0.001
Mechanical ventilation	14.19	6.13 - 35.43	< 0.001
Pancreatic leakage	1.14	0.41 - 2.89	0.786
Intestinal leakage	2.5	0.94 - 6.49	0.06

OR, odds ratio; CI, confidence interval; RAP, recurrent acute pancreatitis; SAP, severe acute pancreatitis; PH, potential of hydrogen; SCr, serum creatinine; ALB, albumin; TBil, total bilirubin; NE, neutrophilic granulocyte; LYMPH, lymphocyte; PLT, platelet; PCT, procalcitonin; Hb, hemoglobin; Concurrent GPB infection, Concurrent Gram-positive bacterial infection.

### Identification of important variables with Boruta analysis

**Figure 1:**
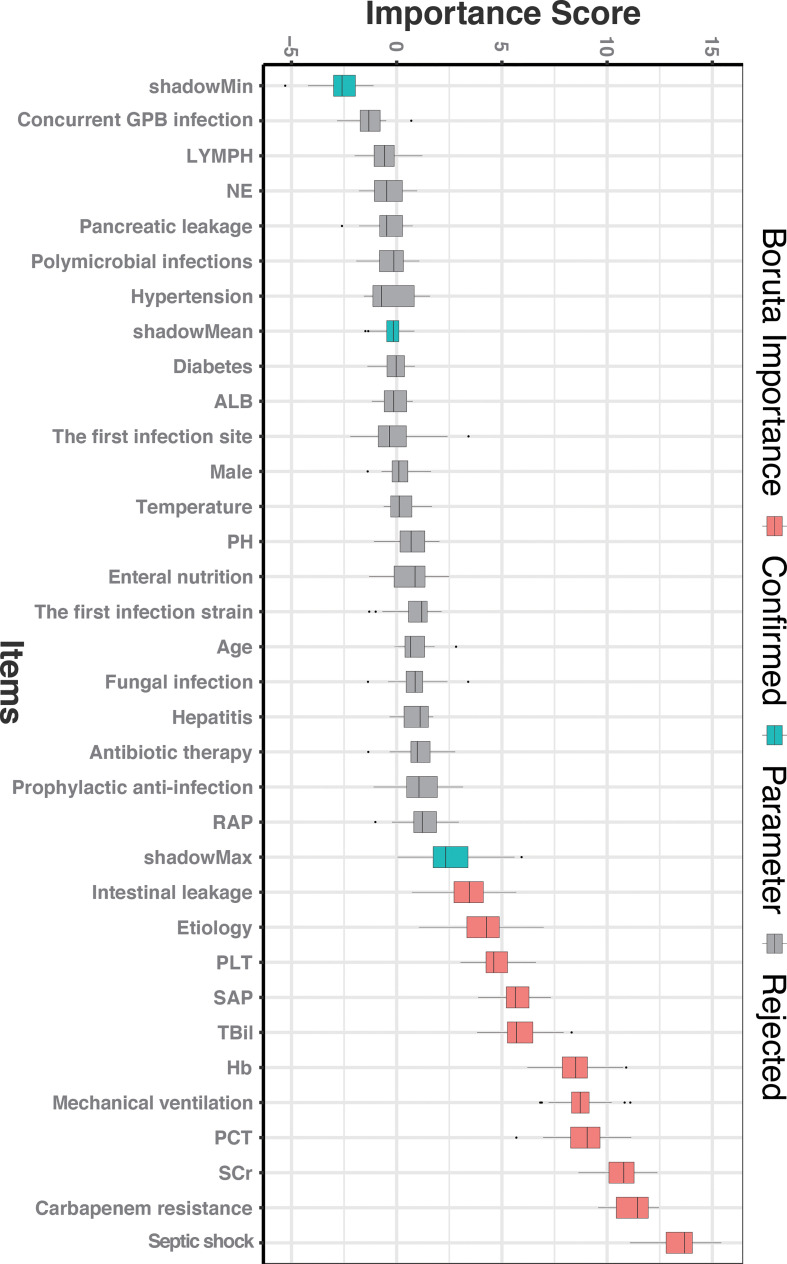
The feature importance in the Boruta feature selection process. The red box represents the feature that is confirmed as important, the green box represents the Boruta parameter, and the gray box represents the feature that is rejected. SCr, serum creatinine; PCT, procalcitonin; Hb, hemoglobin; TBil, total bilirubin; SAP, severe acute pancreatitis; PLT, platelet; RAP, recurrent acute pancreatitis; PH, potential of hydrogen; ALB, albumin; NE, neutrophilic granulocyte; LYMPH, lymphocyte; Concurrent GPB infection, Concurrent Gram-positive bacterial infection.

To obtain all relevant classified features according to importance, 32 clinical characteristics were evaluated using the Boruta feature selection method. The hierarchical graph of feature importance is shown in **Figure 1**. When the feature variable score is above the shadow Max, the feature was accepted; among them, 11 clinical indicators, including SAP, intestinal leakage, etiology, platelet counts, bilirubin, hemoglobin, intubation, procalcitonin, creatinine, carbapenem resistance, and septic shock were important. Not surprisingly, carbapenem-resistant GNB infection and septic shock were significantly associated with the risk of death from moderate SAP. All other variables with an importance score lower than the Shadow max were rejected.

### Establishment of death prediction nomogram

**Figure 2:**
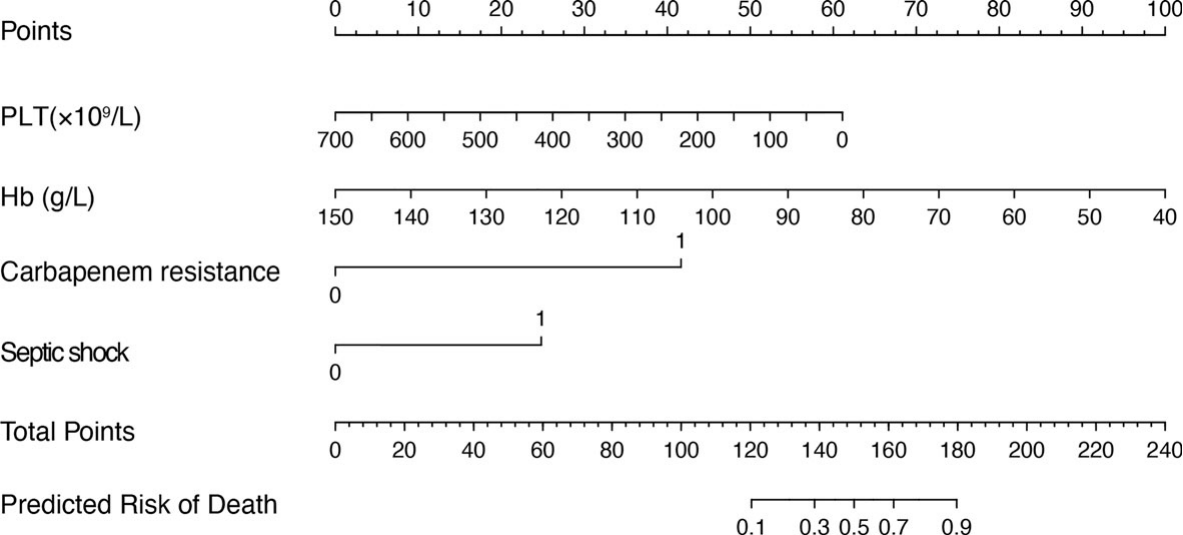
Nomogram for predicting the mortality risk in patients with SAP and GNB infection. To evaluate the risk of mortality *via* the nomogram, draw a line perpendicular to the specific axis of each parameter until it reaches the top line marked “Points”, sum up the number of points for all parameters, and draw a line descending from the axis marked “Total Points” until it reaches the bottom line to determine the predictive risk of death. PLT, platelet; Hb, hemoglobin.

Furthermore, we added the intersection of these 11 variables identified by univariate logistic regression analysis and the 11 variables selected by Boruta into the multivariate logistic regression analysis. Among them, platelets (OR 0.99, 95% confidence interval [CI] 0.99–1.00; p = 0.007), hemoglobin (OR 0.96, 95% CI 0.921; p = 0.037), septic shock (OR 6.33, 95% CI 1.12–43.47; p = 0.044), and carbapenem resistance (OR 7.99, 95% CI 1.66–52.37; p = 0.016) were confirmed as independent risk factors for mortality in patients with moderately SAP and SAP (**Table 3**). These factors were used to construct a multivariate logistic regression prediction model for mortality risk, which was further visualized as a nomogram as shown in **Figure 2**.

**Table 3:** Multivariate logistic regression analysis of mortality based on baseline characteristics in the survival group and death group.

Variables	OR	95% CI	p-value
SCr	1	0.99 - 1.00	0.808
TBil	1.01	1.00 - 1.02	0.093
PLT	0.99	0.99 - 1.00	0.007
Hb	0.96	0.92 - 1.00	0.037
PCT	1.04	0.99 - 1.10	0.143
Carbapenem resistance	7.99	1.66 - 52.37	0.016
Septic shock	6.33	1.12 - 43.47	0.044
SAP	4.86	0.87 - 42.19	0.097
Mechanical ventilation	1.53	0.35 - 6.34	0.556

OR, odds ratio; CI, confidence interval; SCr, serum creatinine; TBil, total bilirubin; PLT, platelet; Hb, hemoglobin; PCT, procalcitonin; SAP, severe acute pancreatitis.

### Verification of the developed nomogram

**Figure 3:**
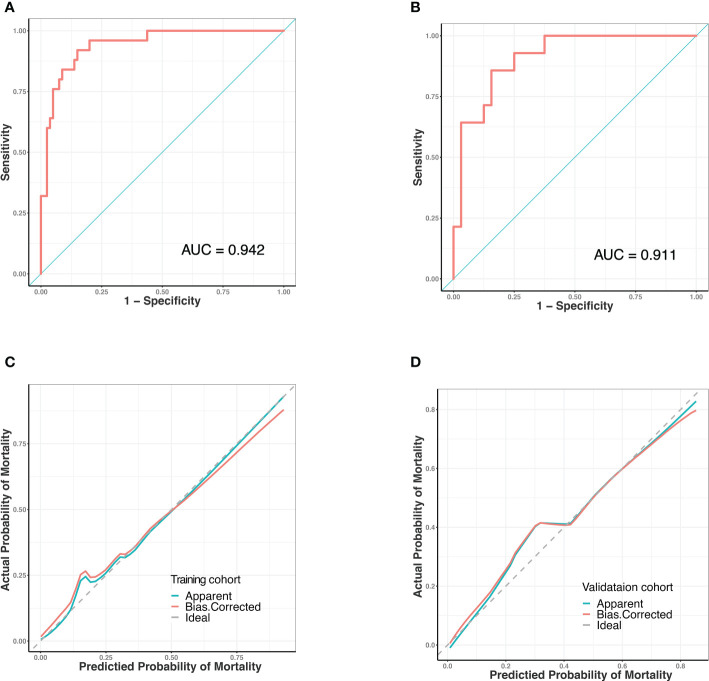
Receiver operating characteristic (ROC) curves and predictive performance of the nomogram for predicting mortality risk in SAP patients with GNB infection. **(A)** ROC curve for the predictive model of the training cohort. Area under the curve was 0.942. **(B)** ROC curve for the predictive model of the validation cohort. Area under the curve was 0.911. **(C)** Validity of the predictive performance of the nomogram in estimating the risk of mortality in the training cohort. **(D)** Validity of the predictive performance of the nomogram in estimating the risk of mortality in the validation cohort. ROC, receiver operating characteristic.

Predictive models were internally validated using the bootstrap validation method. The receiver operating characteristic curves (ROC) for prediction model in training and validating cohorts are shown in **Figures 3A, B**. The nomogram showed good accuracy in estimating the risk of death among patients with SAP who were infected by GNB. In addition, we applied our model to the training and validation cohorts and conducted correlation analysis to evaluate the performance of the nomogram. The calibration plot graphically showed good consistency in the risk estimated by the nomogram and the actual mortality of patients with SAP (**Figures 3C, D**). The sensitivity and specificity of this nomogram for identifying death in patients requiring GNB infection were 96% and 80% in the training cohort and 92.8% and 75% in the validation cohort, respectively.

## Discussion

Local or systemic infection secondary to SAP is the primary cause of exacerbation in the late stage among which GNB ranks the most important causal pathogen while the influence the infection brought to patient prognosis remains unknown. In this study, we conducted a logistic regression analysis and feature selection to identify four variables: (1) septic shock, (2) carbapenem resistance, (3) hemoglobin, and (4) PLT and developed an easy-to-use visual nomogram to predict the risk of mortality in patients with advanced GNB infection in SAP. The nomogram showed good predictive power. To our knowledge, this is the first report of a clinically-applied predictive model for predicting the risk of mortality in patients with SAP and late-stage GNB infections.

In this study, patients with SAP and GNB infection had a mortality rate of 25.8%. In fact, previous study reported a mortality rate of 34.5% for patients with SAP and secondary infections (Tian et al., 2020) and 40% for patients with necrotizing pancreatitis and concurrent infection (Biberci Keskin et al., 2020). The most common etiology of AP was hyperlipidemia (42.4%), which has been reported as the main cause (58.9%) in patients with multidrug-resistant (MDR) pathogen infections (Li et al., 2020). The rate of RAP was 15.2%, which was similar to the previously reported incidence in about 17% (Ahmed Ali et al., 2016). The rate of carbapenem resistance was 45.7%, slightly lower than the ratio of beyond 50% reported by other studies (Chen et al., 2021). The most common site of infection was the pancreas, and the most common causal pathogen was *K. pneumoniae*, which was similar to the previous literature (Wu et al., 2022).

The translocation of intestinal bacteria (namely, intestinal bacteria passing through the intestinal mucosal barrier, and invading the systemic circulation and extra-intestinal organs) could cause damage to extra-intestinal organs, which led to secondary multiple organ failure and eventually to sepsis (Wang et al., 2019). Septic shock was the most serious infectious complication of bloodstream infection. GNB was most likely to elicit inflammatory reactions within the bloodstream when they discharge large amounts of endotoxin-containing membrane blebs (Munford, 2006). In our study, septic shock was found to be an independent predictive risk factor in patients with SAP and Gram-negative organism infection, manifesting as systemic inflammatory response and secondary multi-organ failure. In fact, septic shock was reported associating with higher mortality in patients with AP and multidrug-resistant *K. pneumonia* infection (Devani et al., 2018), which was consistent with our results. Together with the previous study and our findings, patients with SAP and septic shock should receive prompt and rational treatment, not least antibiotic therapy, as explicit guidelines for early initiation of antiseptic shock therapy are available (Permpikul et al., 2019).

It has been reported that carbapenem has been considered as the last-line regimen for AP patients with “suspected” pancreatic infections (Guo et al., 2022). Due to relative resistance to hydrolysis by most β-lactamases, carbapenems are regarded as the most active and potent agents against multidrug-resistant (MDR) gram-negative pathogens with a wide antibacterial spectrum (El-Gamal et al., 2017).However, the inappropriate use of the antimicrobial drugs has led to the rise of the drug-resistant pathogens, which, according to the reporting frequency, are sorted from high to low as Carbapenem-resistant *Enterobacteria* (CRE), Carbapenem-resistant *Pseudomonas aeruginosa* (CRPA), and Carbapenem-resistant *Acinetobacter baumannii* (CRAB) complex (Tacconelli et al., 2018). The overuse of antibiotics in China ranks first in the world; hence, Chinese patients with AP were more susceptible to drug-resistant pathogen infections (Párniczky et al., 2019). Over the past decade, CRE infections have caused high mortality, thus placing tremendous economic burden in the global healthcare system (Durante-Mangoni et al., 2019). The GNB acquired resistance to certain drugs occurs *via* multiple methods: (1) production of carbapenemases, (2) over-expression of some efflux pumps, (3) increase in membrane permeability as a result of special porins loss. (Higgins et al., 2004, Cornaglia et al., 2007, Zhanel et al., 2007, Nikaido and Pagès, 2012). Thus, carbapenem resistance in Gram-negative pathogens poses a special clinical challenge. Some previous studies elucidated that infection caused by CR- or carbapenemase-producing (CP)-GNB likely led to high mortality rates (Cheng et al., 2015, Liu et al., 2016). Jain et al. revealed that MDR bacterial infection was an independent predictor of mortality in patients with AP and infected pancreatic necrosis (Jain et al., 2018), which was consistent with our findings. Together with previous studies, our data supported the idea that close attention should be devoted to the appropriate use of antibiotics. Although some “last line” antibiotics, such as tigecycline, colistin, minocycline, and ceftazidime–avibactam and others might be available for treating CR-GNB under certain conditions (Doi, 2019), and an animal study demonstrated high dosages of aztreonam might work in the future clinical practice (Bellais et al., 2002).Our results showed that prophylactic antibiotic use was not an independent risk factor for death. Nevertheless, the prophylactic use of antibiotics, especially carbapenems, may not be recommended in AP for the prevention of infectious complication.

Platelet count was found as an independent risk factor for death in the patients with SAP and GNB infection. The inflammatory cascade in the AP could cause damage to the vascular endothelium, activate platelet, induce thrombosis, and conversely promote the progression of AP (Beyazit et al., 2012). The abnormality of platelet count could be considered as an assessing indicator of coagulation function and disease severity (Lei et al., 2017). In addition to participating in the hemostasis process, platelets were important players in host defense during infection and battle with invading pathogens *via* various mechanisms, which included interacting with neutrophils or directly with bacteria (Amison et al., 2018). Hematopoietic inhibition, autoimmune attack, and micro thrombosis depletion might contribute to the occurrence of thrombocytopenia (Kelton et al., 1979; Zhang et al., 2020).However, the specific mechanism remains obscure. In a real-world study, thrombocytopenia was also described as a predictor of death in GNB infection, and closely related to ICU mortality (Jonsson et al., 2021; Ni et al., 2022). It implies that more attention should be paid to the platelet count variation in the clinical practice.

The long-term course of SAP leads to accelerated catabolism, increased caloric and nutrient requirements, and in many cases, nutritional deficiencies, which causes an increase in the incidence and mortality of infections. An important and sensitive indicator reflecting nutritional deficiencies was anemia, and severe anemia was associated with poor prognosis in patients with SAP (Ibrahim et al., 2017; Oh et al., 2021). A study of risk factors for mortality in patients with AP also found that lower hemoglobin was associated with an increase in mortality, which was consistent with our conclusion (Popa et al., 2016). Inflammatory responses were found to influence hemoglobin levels through various approaches, including suppressed erythropoiesis, shortened erythrocyte survival, and decreased erythropoietin production (Ganz, 2019). Besides, the endotoxin released by GNB could also damage the vascular endothelium and even cause disseminated intravascular coagulation, resulting in impaired hemostatic function and blood loss (Schrottmaier et al., 2016). Anemia might affect the prognosis of an individual with infection. When hemoglobin was at a low level, oxygen delivered to major organs was reduced, leading to tissue hypoxia, which can ultimately contribute to multiple organ dysfunction in infected patients (Cavezzi et al., 2020). A related study in patients with sepsis further supported our findings (Muady et al, 2016), indicating anemia will lead to a higher risk of increased mortality in patients with infection.

Univariate logistic regression analysis showed that serum creatinine, bilirubin levels, PCT, SAP, and mechanical ventilation were associated with patient mortality, a finding that is consistent with the results of real-world studies, while in our multivariate logistic regression analysis, these several parameters were not independently associated with the risk of mortality. Confounding factors such as platelets, hemoglobin, septic shock, and carbapenem-resistant infections might play an important role in mortality. Multiple organ failure concomitant with septic shock leads to secondary associations between laboratory biochemical markers and mortality. Carbapenem-resistant pathogen infections lead to an increase in the occurrence of mechanical ventilation (Zilberberg et al., 2017). Our results suggest that the above variables did not by themselves directly affect the risk of mortality even though they were closely associated with disease severity. The higher comorbidity burden and greater severity of illness might contribute to wider use of antibiotics in the death group when compared with the survival group.

The purpose of our study was to develop a visual and quantitative nomogram to predict the risk of death in patients with SAP and GNB infections. The application of a nomogram to assess the risk of death in patients with SAP is a new concept. Our predictive model is sensitive and easy to use, enabling efficient and accurate identification in clinical practice. Moreover, the model has good predictive performance. By calculating a total score based on four objective clinical variables (septic shock, carbapenem resistance, platelet counts, and hemoglobin levels), physicians can quantify the mortality in patients with SAP and GNB infections, contributing to more timely and comprehensive diagnosis and treatment.

The calibration curve fits well. The use of this predictive model can provide important guidance for clinical decision making and optimal allocation of medical resources which is expected to improve patient prognosis and quality of life. Using this predictive model to accurately identify patients with high risk of death can enhance clinical symptom warning, optimize the allocation of medical resources, facilitate clinical decision-making, and possibly improve the clinical outcomes of patients with SAP.

Several limitations should be discussed. First, clinical data from patients in the cohort were retrospectively collected; therefore, inherent biases were unavoidable. Second, external and prospective validation in a new large cohort is required to determine diagnostic accuracy and clinical application. Finally, the recapitulative nomogram was used to predict mortality risk for all types of GNB infection, which might temper the efficacy of our nomogram. It would be more precise to expand the sample size in the future study and promote the nomogram according to the specific causal GNB.

## Conclusion

We developed a clinically applicable nomogram for predicting the risk of mortality in patients with SAP and GNB infection. This nomogram enables early and efficient assessment of mortality risk in patients with SAP infected by GNB, which may provide guidance for clinical decision-making.

## Data availability statement

The original contributions presented in the study are included in the article/**Supplementary Material**. Further inquiries can be directed to the corresponding author.

## Ethics statement

The studies involving human participants were reviewed and approved by the Institutional Review Board of Xiangya Hospital, Central South University. Written informed consent for participation was not required for this study in accordance with the national legislation and the institutional requirements.

## Author contributions

Study concept and design: JY. Acquisition of data: JY, WD, WJ, and WH. Statistical analysis: JY. Analysis and interpretation of data: JY and HY. Drafting of the manuscript: HY and JY. Critical revision of the manuscript: PJ. All authors contributed to the article and approved the submitted version.

## Funding

This work was supported by grants from the Fundamental Research Funds for the Central Universities of Central South University (2021zzts0352), and National Natural Science Foundation of China (Grant No. 82170661).

## Acknowledgments

We appreciate the professionalism and compassion demonstrated by all the healthcare workers involved in patient care. We also acknowledge all the patients for their involvement in this study.

## Conflict of interest

The authors declare that the research was conducted in the absence of any commercial or financial relationships that could be construed as a potential conflict of interest.

## Publisher’s note

All claims expressed in this article are solely those of the authors and do not necessarily represent those of their affiliated organizations, or those of the publisher, the editors and the reviewers. Any product that may be evaluated in this article, or claim that may be made by its manufacturer, is not guaranteed or endorsed by the publisher.
