# Supplementary material for: A nomogram for predicting the risk of mortality in patients with acute pancreatitis and Gram-negative bacilli infection
Source: Front Cell Infect Microbiol. 2022 Nov 10;12:1032375. doi: 10.3389/fcimb.2022.1032375 (PMC9685314; doi:10.3389/fcimb.2022.1032375)
Supplement: Supplementary file 1 [file Table_1.docx]

| SUPPLEMENTAL TABLE 1 Baseline characteristics of patients in training cohort and validation cohort. |
| --- |

| Variables (n (%), median (IQR) or mean ± SD) | Overall (n =151) | Training cohort (n = 105) | Validation cohort (n = 46) | p-value |
| --- | --- | --- | --- | --- |
| Male | 107 (70.9) | 75 (71.4) | 32 (69.6) | 0.97 |
| Age, years | 48.44 (12.53) | 48.29 (12.72) | 48.80 (12.22) | 0.816 |
| Comorbidity |  |  |  |  |
| Hypertension | 31 (20.5) | 22 (21.0) | 9 (19.6) | 1 |
| Diabetes | 32 (21.2) | 24 (22.9) | 8 (17.4) | 0.589 |
| Hepatitis | 4 (2.6) | 2 (1.9) | 2 (4.3) | 0.586 |
| Etiology |  |  |  | 0.153 |
| Hypertriglyceridemia | 64 (42.4) | 43 (41.0) | 21 (45.7) |  |
| Gallstones | 39 (25.8) | 23 (21.9) | 16 (34.8) |  |
| Others | 27 (17.9) | 20 (19.0) | 7 (15.2) |  |
| Alcoholism | 18 (11.9) | 16 (15.2) | 2 (4.3) |  |
| Idiopathic | 3 (2.0) | 3 (2.9) | 0 (0.0) |  |
| RAP | 23 (15.2) | 16 (15.2) | 7 (15.2) | 1 |
| SAP | 81 (53.6) | 53 (50.5) | 28 (60.9) | 0.317 |
| Enteral nutrition, days | 3.00 (2.00, 5.00) | 3.00 (2.00, 5.00) | 3.00 (1.25, 6.00) | 0.617 |
| PH | 7.43 (7.27, 7.48) | 7.44 (7.27, 7.48) | 7.42 (7.29, 7.47) | 0.397 |
| SCr (mmol/L) | 66.80 (52.95, 100.75) | 66.00 (52.50, 100.00) | 71.95 (53.20, 104.10) | 0.633 |
| ALB (g/L) | 29.92 (4.76) | 30.12 (4.63) | 29.45 (5.08) | 0.427 |
| TBil (mmol/L) | 15.70 (9.50, 26.85) | 15.80 (10.50, 28.40) | 14.00 (8.93, 24.80) | 0.174 |
| NE (×109/L) | 8.20 (5.60, 12.50) | 8.20 (5.70, 12.70) | 8.05 (5.18, 12.18) | 0.749 |
| LYMPH (×109/L) | 0.90 (0.60, 1.20) | 0.90 (0.60, 1.20) | 0.90 (0.60, 1.20) | 0.681 |
| PLT(×109/L) | 271.99 (132.69) | 276.10 (139.69) | 262.61 (116.02) | 0.567 |
| Hb (g/L) | 88.13 (17.75) | 87.97 (16.78) | 88.48 (19.97) | 0.872 |
| PCT (ng/L) | 1.11 (0.28, 3.98) | 1.19 (0.32, 4.45) | 0.62 (0.22, 3.12) | 0.282 |
| Temperature, ℃ | 39.00 (38.00, 39.50) | 39.00 (38.00, 39.40) | 39.00 (38.52, 39.65) | 0.258 |
| Mortality | 39 (25.8) | 25 (23.8) | 14 (30.4) | 0.513 |
| Carbapenem resistance | 69 (45.7) | 45 (42.9) | 24 (52.2) | 0.379 |
| Septic shock | 26 (17.2) | 18 (17.1) | 8 (17.4) | 1 |
| Mechanical ventilation | 48 (31.8) | 28 (26.7) | 20 (43.5) | 0.064 |
| The first infection site |  |  |  | 0.366 |
| Pancreas (peri) | 98 (64.9) | 67(64.5) | 31 (67.4) |  |
| Bloodstream | 16 (10.6) | 14 (13.3) | 2 (4.3) |  |
| Lung | 28 (18.5) | 17 (16.2) | 11 (23.9) |  |
| Urinary system | 2 (1.3) | 1 (1.0) | 1 (2.2) |  |
| Others | 7 (4.6) | 6 (5.7) | 1 (2.2) |  |
| The first infection strain |  |  |  | 0.983 |
| Escherichia coli | 32 (21.2) | 22 (21.0) | 10 (21.7) |  |
| Klebsiella ozaenae | 45 (29.8) | 31 (29.5) | 14 (30.4) |  |
| Acinetobacter baumannii | 33 (21.9) | 23 (21.9) | 10 (21.7) |  |
| Pseudomonas aeruginosa | 10 (6.6) | 8 (7.6) | 2 (4.3) |  |
| Others | 31 (20.5) | 21 (20.0) | 10 (21.7) |  |
| Polymicrobial infections | 77 (51.0) | 53 (50.5) | 24 (52.2) | 0.988 |
| Concurrent GPB infection | 46 (30.5) | 34 (32.4) | 12 (26.1) | 0.561 |
| Fungal infection | 50 (33.1) | 37 (35.2) | 13 (28.3) | 0.515 |
| Prophylactic anti-infection |  |  |  | 0.426 |
| Wide-spectrum antibiotics | 51 (33.8) | 37 (35.2) | 14 (30.4) |  |
| Carbapenem | 45 (29.8) | 34 (32.4) | 11 (23.9) |  |
| Combined antibiotics | 46 (30.5) | 29 (27.6) | 17 (37.0) |  |
| Antibiotic therapy |  |  |  | 0.114 |
| Carbapenem (high-dose, extended-infusion) | 53 (35.1) | 40 (38.1) | 13 (28.3) |  |
| Penicillins/b-lactamase inhibitors | 29 (19.2) | 19 (18.1) | 10 (21.7) |  |
| Tigecycline | 2 (1.3) | 2 (1.9) | 0 (0.0) |  |
| Carbapenem and tigecycline | 44 (29.1) | 33 (31.4) | 11 (23.9) |  |
| Carbapenem and penicillins/b-lactamase inhibitors | 21 (13.9) | 10 (9.5) | 11 (23.9) |  |
| Carbapenem and sulfonamides | 1 (0.7) | 0 (0.0) | 1 (2.2) |  |
| Tigecycline and polymyxin | 1 (0.7) | 1 (1.0) | 0 (0.0) |  |
| Pancreatic leakage | 25 (16.6) | 20 (19.0) | 5 (10.9) | 0.314 |
| Intestinal leakage | 21 (13.9) | 15 (14.3) | 6 (13.0) | 1 |

IQR, interquartile ranges; SD, standard deviation; RAP, recurrent acute pancreatitis; SAP, severe acute pancreatitis; PH, potential of hydrogen; SCr, serum creatinine; ALB, albumin; TBil, total bilirubin; NE, neutrophilic granulocyte; LYMPH, lymphocyte; PLT, platelet; Hb, hemoglobin; PCT, procalcitonin; Concurrent GPB infection, Concurrent Gram-positive bacterial infection.

.
